# Editorial: Exploring bioactive polyphenols in agri-food wastes: from extraction to biological impact

**DOI:** 10.3389/fnut.2025.1638180

**Published:** 2025-07-01

**Authors:** Francisca Rodrigues, Doretta Cuffaro, Maria Digiacomo

**Affiliations:** ^1^UCIBIO—Applied Molecular Biosciences Unit, MedTech-Laboratory of Pharmaceutical Technology, Faculty of Pharmacy, University of Porto, Porto, Portugal; ^2^Associate Laboratory i4HB—Institute for Health and Bioeconomy, Faculty of Pharmacy, University of Porto, Porto, Portugal; ^3^Department of Pharmacy, University of Pisa, Pisa, Italy; ^4^Interdepartmental Research Center “Nutraceuticals and Food for Health, ” University of Pisa, Pisa, Italy

**Keywords:** polyphenols, bioactive compounds, circular economy, food chemistry, food waste

The sustainable valorization of agro-food waste and by-products represents a critical frontier in advancing the circular bioeconomy. As global food systems face increasing pressure to reduce environmental impact and optimize resource use, the concept of “waste” is being fundamentally reimagined. Agricultural and food production residues, once discarded as low-value biomass, are now recognized as rich sources of bioactive compounds, functional materials, and nutraceutical ingredients (1). This Research Topic compiles a series of innovative studies that explore diverse strategies for transforming agro-industrial by-products into valuable components for food, pharmaceutical, and material applications.

Our objective in curating this Research Topic is to present multidisciplinary approaches that address both environmental challenges and industrial opportunities. These studies exemplify how scientific innovation can unlock the nutritional, functional, and therapeutic potentials embedded in food chain residues, thereby aligning sustainability with technological advancement.

## Advancing agro-waste valorization: optimizing extraction and functional delivery technologies

A critical step in agro-waste valorization lies in the development and optimization of extraction technologies capable of isolating high-value compounds in an efficient, selective, and environmentally responsible manner. Conventional extraction methods often rely on energy-intensive protocols and large volumes of hazardous solvents, limiting their sustainability and scalability. In response to these Research Topics, innovative green extraction approaches have emerged, with microwave-assisted extraction (MAE) gaining particular attention for its ability to enhance extraction efficiency while minimizing chemical usage and processing time (2).

A noteworthy example is provided by Valdés et al., who successfully applied MAE to recover cellulose nanocrystals (CNCs) from almond (*Prunus amygdalus*) shell waste. Their study demonstrated that MAE not only improved the crystallinity and yield of CNCs compared to traditional techniques, but also significantly reduced environmental burdens associated with solvent consumption. The resulting nanocellulose materials hold considerable promise in a wide range of applications, including biodegradable packaging, food coatings, and biomedical devices, illustrating how process optimization can align waste recovery with emerging material demands.

However, extraction is only one facet of a comprehensive valorization strategy. Equally important is the functional delivery of the bioactive compounds, particularly in food and nutraceutical systems where stability, bioavailability, and controlled release are critical parameters. In this context, advanced encapsulation technologies offer a powerful means to protect sensitive bioactives and enhance their performance in target applications (3). This is exemplified in the work by Khazaal et al., who extracted phenolic compounds from sesame seed coat waste and encapsulated them into nanoemulsions for antimicrobial use in milk preservation. Their integrated approach from extraction to application not only demonstrated the efficacy of the phenolics in reducing microbial load, but also highlighted how nanoencapsulation can improve solubility, stability, and bioefficacy.

## Phytochemical variation in agro-biodiversity

The intrinsic biological and ecological factors that regulate phytochemical accumulation are important aspects to consider in the field of bioactive compound recovery from plant sources. Two particularly critical, yet often underappreciated, variables are harvest timing and genetic diversity, both of which have profound effects on the nutritional properties and composition of different plant species and food matrix (4–10).

Dai et al. addressed the influence of seasonal dynamics on bioactive compound profiles in *Rheum officinale*, by systematically analyzing leaf blades at different growth stages. Their study revealed marked temporal fluctuations in key phytochemicals, particularly flavonoids and phenolics, with antioxidant activity peaking at distinct time points during the growth cycle. These findings highlight the potential of timing-specific harvesting as a low-cost yet high-impact strategy to enhance the yield and bioefficacy of plant-derived compounds. Such temporal optimization aligns closely with the principles of precision agriculture and offers valuable guidance for industries engaged in the production of functional foods, supplements, and phytopharmaceuticals.

In the same field, the mini-review by Pérez-Ramírez et al. explored natural variation in nutraceutical composition among wild piquin chili. By comparing flavonoid, carotenoid, and capsaicinoid content across diverse environmental conditions and maturation stages, the authors underscored the significant role of genotype-environment interactions in determining bioactive profiles. This biodiversity-driven approach not only reveals underutilized sources of high-value compounds but also supports the strategic conservation of wild germplasm as a foundation for future nutraceutical innovation. Moreover, their insights may inform selective breeding programs aimed at enhancing phytochemical concentrations for targeted health applications.

## Pharmacological valorization

In recent years, the valorization of agro-food by-products has extended beyond traditional applications in food and materials science into the realm of pharmacology (11–13). This expansion opens new frontiers for the use of agro-waste in addressing complex health challenges, particularly in the development of novel therapies for neurodegenerative diseases. One compelling example of this shift is the work by Wang et al. who explored the neuroprotective potential of bioactive compounds derived from cinnamon leaves.

Their study utilized advanced UPLC-MS/MS techniques to identify key bioactive compounds—such as cinnamaldehyde and various flavonoids—present in cinnamon leaves. These compounds were subsequently encapsulated in nanoemulsions, a cutting-edge formulation technology that significantly enhances the bioavailability, stability, and controlled release of bioactives. In a rat model of Parkinson's disease, the nanoformulated cinnamon leaf extract demonstrated significant improvements in motor function and biochemical markers, providing compelling evidence for the therapeutic potential of agro-food by-products.

This research exemplifies the dual benefits of agro-waste valorization: not only can it address environmental sustainability by reducing waste, but it can also contribute to pharmacological innovation, particularly in the context of neurodegenerative diseases. Furthermore, the application of nanoformulation techniques highlights the importance of advanced delivery systems in improving the efficacy and therapeutic outcomes of bioactive compounds extracted from agricultural residues. Through this integrated approach, agro-waste is not merely seen as a waste product, but rather as a valuable resource with potential far beyond its initial use.

## Conclusions

Together, these studies illustrate a broad yet coherent spectrum of agro-food waste valorization strategies, unified by their commitment to sustainability and innovation. The contributions fall into several thematic categories: technological advancement, exemplified by green extraction methods like MAE for cellulose nanocrystals and the antimicrobial application of nanoencapsulated seed coat phenolics, biodiversity, and temporal insight in composition, as shown in the seasonal analysis of *R. officinale* and in the review of piquin chili variation, and the pharmacological exploration, through cinnamon leaf-derived neuroprotective extracts.

These themes converge on the broader objective of building a circular bioeconomy—one in which agricultural residues are reintegrated into value chains, reducing waste and enhancing resource efficiency. This shift requires not only scientific innovation but also collaboration across disciplines, including food science, environmental chemistry, biotechnology, pharmacology, and material science.
